# Measuring digital transformation stress at the workplace–Development and validation of the digital transformation stress scale

**DOI:** 10.1371/journal.pone.0287223

**Published:** 2023-10-18

**Authors:** Ewa Makowska-Tłomak, Sylwia Bedyńska, Kinga Skorupska, Radosław Nielek, Monika Kornacka, Wiesław Kopeć

**Affiliations:** 1 Faculty of Psychology, Institute of Psychology, SWPS University of Social Sciences and Humanities, Warsaw, Poland; 2 Polish Japanese Academy of Information Technology, Warsaw, Poland; 3 Center for Research on Social Relations, Institute of Psychology, SWPS University of Social Sciences and Humanities, Warsaw, Poland; 4 Emotion Cognition Lab, SWPS University of Social Sciences and Humanities, Katowice, Poland; St John’s University, UNITED STATES

## Abstract

Despite the unquestionable advantages of digital transformation (DT) in organizations, the very process of DT could have an impact on the level of stress of the employees. The negative effects of the digital transformation process can be observed during the implementation of information and communication technologies (ICT) solutions. They are further enhanced by the effects of COVID-19 pandemic, as digital transformation has accelerated to allow for remote work. Herein we distinguish between general stress at the workplace and the very specific type of stress, namely digital transformation stress (DTS). We assumed that this type of stress appears when rapid implementation of ICT solutions is introduced with time pressure and incertitude of further results. To quantify this phenomenon, we developed a new self-report scale—the Digital Transformation Stress Scale (DTSS), measuring employees’ stress stemming from the process of digital transformation in organizations. The psychometric validity of the scale was evaluated in two studies: Study1 conducted at the beginning of COVID-19 pandemic in 2020 (N = 229) and Study 2 in 2021 (N = 558), after a year of mostly remote work. The results confirmed good reliability with Cronbach’s Alpha α = .91 in Study 1 and α = .90 in Study 2 and assumed unidimensional factorial validity of the scale in both studies. All items of the scale had good difficulty and discrimination values evaluated in Item Response Theory, i.e., IRT approach. The scale showed predicted convergent validity as the indicator of the digital transformation stress moderately correlated with general stress at work. Moreover, the assumption that even employees with high ICT skills could be affected by DTS was confirmed. Additionally, the results indicated that digital transformation stress was significantly higher among employees who reported both issues: ongoing digital solutions projects at the workplace and high impact of COVID-19 pandemic on their work. The scale could be used in future work on measuring and counteracting digital transformation stress at the workplace.

## Introduction

Although digital transformation (DT) evokes organizational changes, recent observations suggest that DT deviates from the past organizational changes. Nowadays, changes related to Information Technology (IT) and Information Communication Technology (ICT) are much more generative, malleable and combinatorial in comparison to traditional ones [1, 2]. Many modern digital technologies are becoming ubiquitous and not confined to the boundaries of specific companies or industries, and therefore encompass a wider ecosystem and the demand side [3, 4]. Therefore, digital change becomes more multidimensional and requires both technical and social skills. Furthermore, in contrast to traditional organisational changes, DT can cause far more dynamic changes that can be triggered and shaped by episodic (technological/ICT) outbursts [3, 5]. Digital transformation changes not only the organisations but can also cause the sudden extinction of some business models and the emergence of new digital business models, even in non-IT industries [3, 6–10]. Therefore, digital transformation nowadays seems to be a qualitatively different organisational change than the ones previously observed and examined in the literature [8].

The expected effects of DT are improvements in the work efficiency and effectiveness of organizations [1, 11, 12]. Despite the unquestionable advantages of digital transformation [10, 13, 14], the DT process and improper implementation of digital changes and solutions may cause high pressure [11, 12], work overload [13], hassles [15, 16] and challenges in adapting communication to many employees [17, 18]. These demands in turn may increase stress experienced in the workplace [13, 16] and, in consequence, decrease productivity, commitment [17, 18] and more broadly decrease well-being of employees in the workplace [11, 19, 20].

The recent COVID-19 pandemic has accelerated the digital transformation in a wide range of areas, from business to education [21–23]. The national lockdowns have forced public and private sectors to sharply reorganize daily work into remote and online modes practically almost overnight [22, 24, 25]. In consequence, the negative impact of DT on employees’ psychological well-being has been recognised along with the increase of stress related to the acceleration in introduction of ICT solutions and digital changes in organizations [15, 26–28]. This rapid digital transformation process revealed that negative consequences of DT were not only identified in organizations which were forced to implement digital transformation solutions, but also in IT companies which were responsible for introducing IT and ICT solutions in those organisations [7, 29]. It is important to note that, despite the high IT skills of the employees in the latter organisations, the job demands, namely workload, availability, time pressure, stemming from the rapid digital transformation rose to a level beyond manageable and were perceived as stressful for both groups of employees [30, 31].

This situation has emphasized the paucity of tools and research evaluating employees stages of adaptation to digital solutions implementation process and potential factors that can affect this adaptation, with stress among others [32–34]. Monitoring the level of stress stemming from digital transformation, namely digital transformation stress (DTS), has grown particularly important. In response to this demand, we propose a new self-report scale, Digital Transformation Stress Scale, designed to identify the early signs of the very type of stress, resulting from digital transformation process ongoing in the organization at the time. It may serve as a monthly screening test to identify those employees who themselves perceive to be affected by this type of stress in order to prevent potential damage the very stress can cause at the personal level, and consequently, to the organisation.

### Job stress and its measurement

Nowadays, job stress is by far the most significant source of stress for adults and it has been escalating over the past few decades [35–37]. Therefore, its identification at an early stage is an important aim in the workplace to avoid prolonged and negative consequences limiting work satisfaction, motivation, and well-being of employees, as well as potential resignation from work [38–40].

According to the literature and research, stress is a nonspecific response of a person to specific, individually assessed stressors [35, 41]. In the transactional model of stress proposed by Lazarus and Folkman [42] stress is defined as “a particular relationship between the person and the environment that is appraised by the person as taxing or exceeding his or her resources and endangering his or her well-being” [42]. Following this approach, subjective perceptions of an individual appraisal of the stressor as threatening, resulting with anxiety, discomfort, emotional tension, and difficulty in adjustment, are the crucial components of the stress [35, 41, 42]. Although there is no single definition of stress that is universally accepted [35, 43, 44], these two elements: subjectivity of stress experience and insufficient resources to cope are the most commonly shared elements in all the existing definitions [35, 42, 45, 46].

These two definitional elements are the basis of a commonly used scale assessing perceived stress, namely the Perceived Stress Scale [41], present in the literature and practice in several versions of different length, starting from the 14-item PSS-14 through the most popular 10-item PSS-10 [41, 47, 48] up to the shortest, 4-item version, i.e., PSS-4 [47, 49]. Based on stress-appraisal theory [42], all versions of PSS evaluate the degree to which an individual (here employee) has experienced distress and negative feelings stemming from perceiving life situations and the feeling that they cannot cope with a specific situation or stimulus [41, 43, 50]. The PSS is composed of items that measure how uncontrollable, unpredictable, and overloaded individuals evaluate their life situation [41] in different contexts, e.g. the workplace. Currently, the PSS-4 is commonly used and recommended in situations where a very short scale is required, like Internet surveys or telephone interviews [41, 47]. The PSS-4 is the scale with the combination of good psychometric properties with the ease of administrating in the applied settings.[47, 49] In addition to the above-mentioned definitional components of stress, there is also agreement between researchers that the identification of specific stressors is associated with many difficulties, because people often misattribute their feelings of stress to a particular source when that stress is actually due to another source [41, 44]. Therefore, more promising in assessing the level of stress is rather to evaluate a context area, which may represent a potential source of psychological harm to the individuals. One of such contexts is job stress; i.e., stress perceived by the employees in the context of workplace. With reference to this context, stress is defined as a particular individual’s subjective awareness or feeling of personal dysfunction as a result of perceived conditions or events occurring in the work settings [50]. Similarly to the definition of general stress [51, 52], subjective appraisal is identified as evoking an emotional reaction to stress and different coping strategies [42]. Consequently, because of the importance of subjective appraisal, the occurrence of the same situation may be perceived by one employee as stressful, and by another as not [42]. Therefore, self-descriptive measures of stress, asking participants about their subjective experience are very common in the literature and practice [14, 41, 43, 53]. Almost all existing measures of stress concentrate on these subjective feelings of employees, more commonly on emotional response to stimuli, such as psychological distress [54], which was confirmed in multiple studies as the first sign that environmental job demands exceed one’s perception of the ability to cope [35, 50, 53, 55], or a sign of perceiving the situation at work as threatening [54, 56–58]. One of the measures used to assess the level of stress in the workplace was the Perceived Stress Scale (PSS) [41], although it offers only a proxy for the perception of stress at the workplace. The PSS is aimed at measuring global perception of stress, without differentiating between life and work stress. These two types of stress are interrelated, and the PSS has often been, and continues to be, used as a measure of work-related stress due to the scale’s universality and the ease of relating the measurement to the context of, for example, the workplace. However, more specific job stress measures were also proposed [36, 59–61]. Further measures of stress at work are multidimensional scales, such as Job Content Questionnaire (JCQ) [60], Effort-Reward Imbalance Questionnaire (ERI-Q) [62], or Occupational Stress Indicator (OSI) [63]. All of the scales indicated to have good psychometric properties in different cultural contexts [64]. However, they are aimed at measuring specific antecedents of work related stress, such as e.g., decision latitude, psychological demands at work, social support in JCQ, lack of fairness at work in ERI-Q, work management, work schedules and work rules, among others, in OSI [14, 62, 63]. Although these factors were presented as significant predictors of stress at work [53, 65, 66], their high level does not automatically translate into a high level of stress of the employees, as research on organizational change indicated (18). As Mukerjee at al. suggested [16], employees may react to organisational changes with stress when they perceive job demands as hindrance instead of challenge. In their model of coping with stress, researchers also noted that concentrating solely on job demands cannot explain what factors enact one of these two perceptions of stress [40, 54, 67].

The role of job demands in predicting dual ways of coping with stress was defined in the Job Demands-Resources (JD-R) model [68, 69]. This approach assumed that employees’ stress in the workplace increases when the job demands, such as: high work pressure [68, 70], workload [14, 71], hassles [14], lack of control [14, 60] increase, but may be buffered by the employee’s resources utilized to balance these demands [68, 70]. The key components of individual adaptive capacity, identified in the context of job stress as personal resources are occupational self-efficacy [72], organizational-based self-esteem [73], and optimism [74].

While JD-R theory explores the role of demands-resources trade-off, the conservation of resources (COR) theory (55,56), being more general, shifts the emphasis from the balance between the job demands and employee’s resources into potential threat of losing valuable resources. In this approach, the psychological stress is defined as an individual’s reaction to an environment in which valuable resources are threatened. The COR theory underscores the critical role of gaining, retaining and protecting the valuable resources, namely personal, social and material resources in a stressful situation. Following this assumption, stress occurs when important resources are either threatened with loss, lost or not gained. Additionally, the basic principle of COR also states that resource loss is disproportionately more salient than resource gain [75], with gain cycle developing slower than loss cycle in individuals and organisations. At the organisational level, developing new resources such as new skills of employees to prepare for an upcoming change is a slower cycle than a potential loss of skills and competencies when this change has to be rapidly implemented [56]. Additionally, in occupational context, threat of losing skills and competencies and the following threat of losing a job position may lead to higher stress, even in highly competent employees.

One of the sources of job stress is organisational change [76], which has nowadays become a permanent element of occupational environment predicting the organisation sustainability in changing economic, social and ecological surroundings in many organisations [77]. In the context of organisational change, the dual model of coping with stress [16, 78], in line with the appraisal theories of stress [42], states that primary appraisal is important to identify the situation as stressful [41, 42]. Then, an individual evaluates their resources to cope with this difficult situation. CoR theory [45, 75] provides a significant theoretical background in defining these resources and their interplay in the context of organisational change [38, 75]. This theory proposes that among primary resources there are material resources, energetic resources (e.g. time), conditions (e.g. seniority) and personal resources (e.g. individual’s traits). The dual model of coping with organisational change underlies the unique role of two distinct types of resources important in this context, namely resilience of the employees, and perceived organisational support. They both determine the ways of coping with the stress evoked by organisational change (proactive or preventive), and predict commitment to change [26, 79, 80].

### Digital transformation stress (DTS)

Based on previous research on job stress [14, 37, 69], we posit that the way of implementing ICT solutions in the organisation may be an important source of stress at workplace. This specific context—namely digital transformation, characterised by specific job demands and buffered by specific resources—should be distinguished from the general job stress, and technostress. According to research on organisational change [81, 82], we aimed to distinguish stress related to change as a consequence of ICT implementations and stress related to improper implementation process of this change. Moreover, we assume, following the Job Demands-Resources model of stress, that growing job demands and lack of important resources (e.g. technical and social support), evoked by digital transformation in the workplace, determine the employees’ negative emotional reactions to changes taking place in the organization during the implementation of IT solutions and new technologies [14, 56, 75, 83].

Following the conservation of resources (CoR) theory [54, 75], we also predict that some employees’ resources e.g., competencies and skills, previously pivotal for effective work, may become less adequate in a new situation, due to changes in ICT solutions. As a result, in the continuous process of digital transformation, individuals could experience negative emotions, distress, a decreasing sense of influence or control over technological changes taking place in the organization, and threat of losing their position or even their job [26, 75, 84, 85]. These theoretical assumptions provide substantial theoretical background to the construction of Digital Transformation Stress Scale.

In this paper, we distinguish digital transformation stress from technostress [27, 86, 87]. Technostress is defined as stress experienced by the individual due to their inability to adapt to new technologies because of the low level of competencies indispensable for taking advantage of modern Information Systems (IS) [5, 21]. Nowadays, many employees understand that ICT solutions and digitization are very important for organizations’ competitiveness and effectiveness [1, 9, 22] and their general attitudes and reaction to new technology are very positive [88, 89]. However, the sharp and improper implementation of new ICT solutions may increase job demands placed on employees as well as threat of losing their significant resources. In consequence, both of the above may increase job stress [89, 90], even among those employees who are highly competent in ICT [30, 89]. Therefore, we named this type of stress, stemming from digital transformation, as perceived digital transformation stress (DTS). In contrast to technostress, we assume that the DTS arises not because of negative attitudes to new technology *per se*, or lack of ICT skills or competencies, but because of the occurrence of a set of factors such as: 1) an improper way of implementing digital solutions and changes in workplaces [91], 2) unfit DT project management, 3) an increase in ICT demands, 4) incertitude of professional future evoked by the globality of the change and potential threat of losing valuable resources. Finally, 5) the DTS may also be related to the stress resulting from organizational changes due to digital transformation [3, 84, 92]. Therefore, DTS is a broader and more complex concept in comparison to technostress and stress related to organizational changes, due to the many factors that may cause it [55, 88, 89].

Although existing measures of technostress have contributed significantly to understanding the antecedents and consequences of stress related to information technology, they were generally not designed to evaluate stress caused exclusively by digital transformation [14, 27, 33, 86, 88]. Similarly, measures proposed in the context of organisational change are not aimed to differentiate digital transformation stress, experienced nowadays by employees in the context of COVID-19 pandemic from stress evoked by general or traditional organizational changes [84, 92].

Distinguishing digital transformation stress from other types of occupational stress is important to allow organizations to address and mitigate the consequences of the introduction of technological stressors in the workplace context [88]. Hence, there is a need to create dedicated psychometric tools to measure perceived stress due to DT process, which will enable data scientists and researchers to explore root causes of this type of stress [55, 88, 93, 94] and find ways to alleviate or address it for the benefit of employees who suffer from it, as well as the organizations undergoing digital transformation [55].

### The development of the Digital Transformation Stress Scale

We developed the scale evaluated in this study to provide a short scale measuring perceived digital transformation stress–the Digital Transformation Stress Scale. The DTSS serves as a psychometric tool targeted to measure stress, caused by the style and manner of ICT tools’ implementation and management, related to time pressure, high workload, and expectations of high efficiency in the context of DT [14, 15, 19, 26]. Since our goal was to create a screening test for DTS, which, together with the sentiment analysis tool [89], set a complex instrument for perceived stress monitoring during DT projects, at intervals of short periods (4–6 weeks) [41, 47], we assumed a brief (few items), one-dimensional scale from the beginning.

We adopted here similar pragmatic approach as Marcatto et al. [79] and we proposed a small set of items, based on two scales assessing perceived stress, i.e., the Perceived Stress Scale [41] and the Perceived Stress at Work [95]. In both scales participants are asked to describe their feelings during the previous month to capture a relatively recent experience in their lives (in PSS) or at the workplace (in PSS-W). Both scales evaluate the degree to which an individual (or employee) has perceived life (of work) as unpredictable, uncontrollable, and overwhelming during the previous month.

Based on Parker, DeCotiis [50] we concentrated on measuring the DTS based on the concept of stress limited to an emotional response to stimuli that may have dysfunctional psychological or physiological consequences. Here, these stimuli were related to the ongoing process of DT in an organization. As people often misattribute sources of stress [41, 50], we decided to construct a scale containing direct inquiries about the perceived stress in the context of the digital transformation, e.g. the process of implementing ICT projects. In designing DTSS, we also assumed that the scale may be practically used as a tool to monitor the level of stress during DT process, in repeated measures design. Therefore, we decided to refer to PSS [41] in order to design our scale because it was proved that it can tap perceived stress fluctuation across different measurement points [41, 43, 55]. We asked our participants to describe their DTS in the previous recent months, because prior studies on the PSS showed that the predictive validity of the scale is expected to decrease rapidly after four to eight weeks [41]. Therefore, the perceived DTS measurement could be retested after that period and any changes could be observed. For the same reason, we wanted to obtain short feeding time (up to a few minutes) and ease to score.

Firstly, we prepared the initial list of 20 proposed items, describing symptoms of stress in the context of digital transformation [83, 96]. Accordingly, in our approach we used similar to PSS phrases referring to the sensations and the frequency of their occurrence, i.e., “*how often have you felt*” [41], similarly referring to previous month (or 4 weeks). However, the content of the items we contextualized to the situation of the digital transformation process, on two theories i.e., Stress appraisal [42] and CoR [75] Theories. According to Lazarus & Folkman’s (1984) stress-appraisal theory and Cohen (1983), we aim to capture whether the employee perceives the situation in the context of work, during the DT and/or ICT implementation, as stressful [41, 42]. Therefore, some items were designed to evaluate negative emotions, (i.e., upset in the first item, irritated in the second item and annoyed in the fourth), which could appear in DT process at the measurement moment. Whereas stress appraisal theory is limited to the assertion that what is perceived as stressful is what is stressful [41, 42, 75] therefore we designed some items we based on CoR theory [54, 67] Thus, two items refer to sense of control or influence: the third: “*How often have you felt that you had no control over ICT changes connected with new procedures and your tasks*?*”* and the fourth: *“How often have you felt annoyed because of new work tasks/rules in connection with system/program changes whose implementation you had no influence on”*. Another item (the sixth one) refers to lack of competencies and skills, i.e., specific personal resources [75] used to deal with ICT demands during ICT solution implementation: “*How often have you felt that your competences and skills were insufficient to be proficient in new IT tools implemented at your workplace*?*”*

Secondly, each proposed item was discussed with experts working in occupational psychology, financial and IT sector employees, and PhD candidates with the aim to optimize the initial list of items for further assessment [96]. Thirdly, the items were examined by the group of six competent judges (psychologists, experts in work and stress psychology), who, based on the DTS definition, on a separate questionnaire, independently evaluated the adequacy of each item. The item ratings were given on a five-point scale—from 1 to 5, where 1 meant that the item did not concern any aspect of DTS and 5 meant that the item was well aligned with the different symptoms of DTS. We used the intraclass correlation coefficient (ICC) to measure inter-rater agreement between judges [97–99].

For above mentioned (and practical reasons), from the beginning, our aim was to develop a brief and less time consuming scale [41, 47, 100, 101], possible to use as a screening test at the workplace, which meant it had to be quicker to complete [41, 47, 101]. Following evidence showing that a short form of the Perceived Stress Scale PSS-10 [41, 48, 102] obtained better psychometric properties than a longer version [47, 48, 103], we decided to select a very limited number of items, with the highest ratings of competent judges and with the high agreement of these ratings. Based on these criteria, we selected 6 items to the final DTSS, with ratings higher than 4.0 and with ICC = .82, *p* < .001.

We evaluated preliminary psychometric properties, namely theoretical validity, in our previous study aimed at developing and evaluating the tool for automatic detection of DT stress [89]. In that study, we used the Digital Transformation Stress Scale (DTSS) and we indicated significant associations between the level of self-reported digital transformation stress with the number of emotional markers evaluated based on sentiment analysis of issues (digital, technical, system problems) registered by employees and sent to a dedicated application defined as the help-desk [89].

Additionally, in our previous study aimed at developing internet intervention addressing DTS [55], we confirmed predictive validity of the DTS by presenting the relation between digital transformation stress, measured by DTS and job burnout, job self-efficacy and negative emotional attitudes to digital transformation [55, 104]. The level of DTS was negatively related to self-efficacy, negative emotional component of attitude towards DT, negative cognition component of attitude towards DT, and positively correlated to both burnout dimensions (with moderate association to exhaustion, and a bit weaker to disengagement). As predicted, digital transformation stress was weakly related to general positive attitudes toward digital change in the organisation [55]. Both studies confirmed that DTSS may serve as a good theoretical and predictive validity.

### Aim of the studies

The central aim of our research is to validate psychometric properties of the Digital Transformation Stress Scale (DTSS), which assesses perceived digital transformation stress defined in terms of employees’ emotional response to the process of digital transformation in the workplace. To fulfil the general aim of this research, we ran two separate studies. In Study 1, we examined the initial psychometric properties of the DTS scale: factorial validity using the principal component analysis, and reliability of the scale evaluated by the Cronbach’s alpha.

In Study 2, we evaluated more advanced psychometric properties: internal structure, convergent and criterion validity, and similarities or differences between items in item functioning. We re-evaluated the assumed unifactorial structure of the scale in confirmatory factor analysis (CFA) in the structural equation modelling approach. We also assessed items functioning in Item Response Theory approach [105], to evaluate its similarities in difficulty and discrimination.

Following the results of Study 1, in Study 2, we continued to evaluate convergent and criterion validity [83, 106] presented also in our previous study [89], where we had indicated a significant association between the occurrence of negative emotion markers in sentiment analysis of helpdesk tickets in one specific organization, with digital stress level assessed using DTSS [89]. Corroborating the findings of the previous research on digital transformation stress, in Study 2 we examined the association between perceived digital transformation stress and general perceived stress at work, assuming that these two types of stress should be related, although not the same [55, 89]. Moreover, in the second study, based on the assumption that digital transformation stress does not stem from the lack of ICS skills, we also examined the association between digital transformation stress and self-assessed ICT skills. In addition, we predicted that employees working in organizations with an ongoing IT implementation project might experience a higher level of perceived digital transformation stress. Furthermore, assuming the COVID-19 pandemic caused acceleration of DT projects in organizations, we compared the level of perceived digital transformation stress in two groups of employees: those who declared that their professional life was affected by COVID-19 pandemic and those who declared they were not affected.

## Study 1

In Study 1, we evaluated the preliminary psychometric properties of DTSS: factorial structure and internal consistency in the sample of employees. We conducted the exploratory factor analysis to study the factorial structure of the scale and we evaluated the scale reliability using Cronbach ‘s Alpha.

### Participants and procedure

The participants constituted a sample of 229 adults (136 women, 75 men, 18 individuals not indicating their gender). All participants were professionally active, working in a range of diverse occupations (e.g., accountants, business analysts, financial analysts, teachers, IT specialists, and managers). A large majority of the participants (82%) had completed higher education and held a full-time job. Most of the participants were between 36 and 45 years old (36.7%). More than 50% of the participants declared their work experience to be over 10 years and nearly 36% of participants declared work seniority between 20 and 30 years. The majority of the participants used ICT in their daily work. The study was conducted in two slots, between the end of December 2019 and February 2020, and after the end of the national lockdown, between mid-June 2020 and the end of September 2020. A more detailed description of the sample is presented in Table 1.

**Table 1:** Sociodemographic information on the participants in Study 1 (n = 229).

Sociodemographic information about the sample (Study 1)						
Statistics	N	%	Female (N)	Female (%)	Male (N)	Male (%)
Total sample	229		136	57%	75	39%
Age						
18–25	5	2.2%	4	2.9%	1	1.3%
26–35	47	20.5%	30	22.1%	17	22.7%
36–45	84	36.7%	52	38.2%	30	40%
46–55	65	28.4%	43	31.6%	22	29.3%
56–65	10	4.4%	7	5.1%	3	4%
over 65	2	0.9%	0	0%	2	2.7%
Degree						
Middle school or lower	24	11.3%	11	8.1%	13	17.3%
University degree	189	82.5%	125	91.9%	62	82.7%
Job seniority						
up to 1 year						
1–3 years	11	5.1%	1	0,7%	1	1.3%
3–10 years	29	12.7%	9	6,6%	10	13.3%
10–15 years	36	15.7%	19	14%	16	21.3%
15–20 years	42	18.3%	27	19,9%	15	20%
20–30 years	82	35.8%	52	38,2%	27	36%
over 30 years	15	6.6%	9	6,6%	6	8%
Job position						
Independent, self-employed	3	1.3%	3	2.2%	0	0%
IT Specialist	3	1.3%	0	0	3	4%
Manager	76	33.2%	40	29%	36	48%
Operational position	32	14%	27	19.9%	4	5.3%
Specialist, analyst, accountant	32	14%	24	17.6%	7	9.3%
Teacher	16	7%	11	8.1%	5	6.7%
Other	51	22%	30	22%	20	26.7%

We mainly recruited the participants via social media, in particular LinkedIn and Facebook, as well as Messenger and WhatsApp. We also recruited participants using the snowball technique through contacts in various organizations from educational and business sectors. Because the study was aimed at employees who use ICT solutions at work, we asked a few Human Resources (HR) managers to send employees an email invitation to the study with a link to the survey. Participants accessed the survey by clicking on the link in the newsletter or email. All data was collected online via Google Forms and the organizational version of the Qualtrics platform, under the university license.

The study was conducted in compliance with ethical standards adopted by the American Psychological Association (APA 2010). The research protocol (with all text contents and compliance with the GDPR) was approved by the Ethical Committee of the University (number of decisions: 47/2020, 50/2020). Accordingly, prior to participation, all participants were informed about the general aim of the research and the anonymity of their data. After marking informed consent to the study, the questionnaire was activated. Participation was voluntary, and participants did not receive compensation for taking part in the study.

### Measures

**The Digital Transformation Stress Scale** (DTSS) consisted of 6 items. The participants were asked to indicate on a five-points scale (1 = Never; 5 = Very often) the frequency of perceived stressful situations concerning the ICT implementation which they experienced during the last four weeks. An example of such an item is “How often have you felt annoyed because of new work tasks/rules involved with the system/program change which you had no influence on?” All items of the DTSS with their translation into English are presented in Table 2. The general indicator of digital transformation stress was prepared by averaging the answers of the participants.

**Table 2:** Descriptive statistics and factor loadings of the Digital Transformation Stress Scale (DTSS) in Study 1 (N = 220).

	Item–English version	Item–Polish version	M	SD	Factor loadings
DTSS	General indicator (average)		2.71	0.85	-
Item 1	How often have you felt upset in connection with new ICT programs/systems?	Jak często czułeś/czułaś się wyprowadzony/a z równowagi, w związku z wdrażanym nowym oprogramowaniem/systemem?	2.90	1.03	.87
Item 2	How often have you felt irritated in connection with new ICT solutions which have affected your professional duties/tasks?	Jak często czułeś/czułaś się zdenerwowany/a w związku wdrażanymi rozwiązaniami informatycznymi (np. nowy system, oprogramowanie), które wpływają na obowiązki służbowe?	2.93	1.06	.86
Item 3	How often have you felt that you had no control over ICT changes connected with new procedures and your tasks?	Jak często miałeś/aś uczucie, że nie ma kontroli nad wprowadzanymi zmianami informatycznymi lub technologicznymi w pracy, powiązanymi z nowymi procedurami i zakresem zadań?	2.84	1.04	.84
Item 4	How often have you felt annoyed because of new work tasks/rules in connection with system/program changes whose implementation you had no influence on?	Jak często denerwowałeś/aś się z powodu nowych zadań/zasad w pracy, w związku ze zmianą systemu/programu, na których wdrożenie nie miałeś/aś żadnego wpływu?	2.90	1.00	.83
Item 5	How often have you felt that what was expected of you due to technological or IT changes was too much for you, to the point where you couldn’t cope with it?	Jak często miałeś/aś uczucie, że postawione przed Tobą wymagania, w związku ze zmianami technologicznymi lub informatycznymi, przerastają Cię i że sobie z nimi nie radzisz?	2.35	1.01	.82
Item 6	How often have you felt that your competences and skills were insufficient to be proficient in new IT tools implemented at your workplace?	Jak często miałeś/aś uczucie, że Twoje kompetencje lub umiejętności są niewystarczające do obsługi nowych narzędzi IT, wdrożonych w organizacji, w której pracujesz?	2.34	1.05	.74

*Note*: DTSS = Digital Transformation Stress Scale

**Socio-demographic information**: participants were asked to indicate their age range, seniority level, gender, education level, occupation, and position in their current job.

### Results

#### Descriptive statistics

Descriptive statistics for all items and the general indicator of digital transformation stress are presented in Table 2. Inspection of descriptive statistics indicated in Table 2 showed that the general level of digital transformation stress was rather moderate with the mean close to the middle point of the scale. The variability was close to one point of the scale.

#### Factorial structure and reliability of the DTS scale

We started the psychometric evaluation of the DTS Scale with a principal component factor analysis with oblique rotation in IBM SPSS Statistics 27.0 to examine the number of factors which can be identified. Three criteria were used to identify the number of factors: theoretical assumptions, the scree test and the eigenvalue greater than one criterion. Loadings were interpreted using cutoffs proposed by Comrey and Lee [107]. Factor loadings higher than or equal to .45 are relevant, loadings higher than or equal to .55 are good, and loadings higher than or equal to .63 are very good [83, 107, 108].

First, data were screened to determine the sampling adequacy using the Kaiser-Meyer-Olkin measure and the KMO test was equal to .85 with *p* < .001, indicating good sampling adequacy [109, 110]. Based on the criteria described above, in the factorial analysis of the DTS scale one factor was extracted and the results confirmed the predicted one-dimensional structure of the scale. The one-dimensional structure explained over 68% of the total variance with six items. All factor loadings of the items of Digital Transformation Stress Scale (DTSS) were high, with the highest being: .86 for item 3 and 1 the lowest: .74 for item 6 (see Table 2). The reliability of DTSS was high, with Cronbach’s Alpha α = .91.

### Discussion and conclusions

The main aim of the first study was to conduct an initial psychometric evaluation of Digital Transformation Stress Scale (DTSS). We examined the preliminary factorial validity and reliability of the tool. The results confirmed the good reliability of the DTSS and assumed unidimensional structure of the scale. The factor loadings of all items developed in the DTSS were moderate or high, therefore there was no need to eliminate any of the items from the Digital Transformation Stress Scale. In conclusion, the results of Study 1 provide support for the assumed structure and internal consistency of the DTSS in the sample of Polish employees. In the next study, we decided to examine the DTSS factorial structure in confirmatory factor analysis and to test theoretical validity of the DTSS by presenting its associations with other variables, e.g., general stress at work.

## Study 2

The main goal of Study 2 was to conduct a more advanced psychometric evaluation of the Digital Transformation Stress Scale (DTSS). We decided to conduct confirmatory factor analysis (CFA) for DTSS. Following theoretical assumptions and the results of Study 1, we predicted a unidimensional structure of the scale. Again, as in Study 1, we examined reliability using Cronbach’s Alpha statistics. To extend the information about the psychometric properties of the DTS scale we conducted Graded Response Polytomous IFA-IRT models for assessment for the extent to which a single latent trait could predict the pattern of associations among these 6 items. The GR model is often used when response data are ordinal, with Likert-type responses [111]. This model is an extension of the dichotomous two parameter logistic IRT model. We also aimed to examine the convergent validity of the scale by presenting the relationships of digital transformation stress with perceived stress at work. We predicted that a higher level of digital transformation stress, measured with DTSS, would be associated with a higher level of general stress. We also tested the relationship between ICT skills and digital transformation stress, and we predicted a rather weak, if any, correlation between these two variables. To test theoretical validity, we also compared digital transformation stress between two groups of workers: affected and unaffected by COVID-19 pandemic. In the same vein, we analysed the differences between employees working in organizations with and without ongoing digital implementation.

### Participants and procedure

The participants of the second study constituted a sample of 558 adults, where 245 were female and 313 male. All participants, except one, were professionally active; most participants have experienced working remotely (n = 335, 60%), whereas 223 participants declared not to have worked remotely at all (40%). The structure of participants comprised of a range of diverse occupations like accountants, business analysts, financial analysts, teachers, IT specialists, and managers, but also engineers, receptionists etc. Majority of the participants had a master’s degree or above: 305 (54%) and 204 (37%) had a bachelor’s degree. Only 36 (6%) participants had the education equal to or lower than middle school. The average age in the sample group was 43.6. The youngest participants were 20 years old and the oldest and professionally active were 69 years old. Most participants were between 40 and 49 years old (183, i.e., 33%), and between 30 and 39 years old (167, i.e., 30%). We grouped the professional occupation declared by the participants into seven job position categories, similarly to Study 1. A more detailed description of the sample is presented in Table 3.

**Table 3:** Sociodemographic information on the participants in Study 2 (N = 558).

	N	%	Female (N)	Female (%)	Male (N)	Male (%)
Sample size	558	-	245	43.9%	313 (56.1%)	56.1%
Age (in years)	*M* = 43.44 (*SD* = 10.71)		*M* = 41.52 (*SD* = 10.99)		*M* = 43.17 (*SD* = 10.45)	
Seniority (in years)	*M* = 18.90 (*SD* = 10.60)		*M* = 17.55 (*SD* = 10.43)		*M* = 19.95 (*SD* = 10.60)	
Remote Work N(%)	335	60%	153	62.4%	182	58.1%
Education (N (%)):						
Primary	3	0.5%	1	0.4%	2	0.6%
Vocational	33	5.9%	8	3.3%	25	8.0%
Secondary	204	36.6%	90	36.7%	114	36.4%
Studying	13	2.3%	7	2.9%	6	1.9%
University degree	305	54.7%	139	56.7%	166	53.0%
Job position (N (%))						
Independent, self-employed	23	4.1%	15	6%	8	3%
ICT specialist	18	3.2%	4	2%	20	8%
Manager	75	13.4%	27	11%	48	20%
Operational position	156	28.0%	102	42%	133	54%
Specialist, analyst, accountant	104	18.6%	55	22%	46	19%
Teacher	33	6.0%	16	7%	15	6%
Others	143	26%	26	11%	43	18%
Self-Assessment ICT Skills M (SD)	*M* = 3.44 (*SD* = 0.88)		*M =* 3.35 (*SD* = .90)		*M* = 3.52 (*SD* = .86)	

The participants were recruited to the study by a professional research agency. All data was collected in online mode only.

The research protocol (with all text contents and compliance with the GDPR) was approved by the Ethical Committee of the SWPS University of Social Sciences and Humanities, Warsaw, Poland, number of decisions: 47/2020, 50/2020, 3/2021, 8/2021. The study was conducted in compliance with ethical standards adopted by the American Psychological Association (APA, 2010). Accordingly, prior to participation, all participants were informed about the general aim of the research and the anonymity of their data. The questionnaire was activated only after the participants declared their informed consent to the study. Participation was voluntary, and the participants did not receive compensation for their participation in the study.

### Measures

**The Digital Transformation Stress Scale** (DTSS) is a self-report scale consisting of six items. All items are presented in Table 4. The participants were asked to indicate on a five-points scale (1 = *Never*; 5 = *Very often*) the frequency of perceived stressful situations concerning the ICT implementation which they experienced during their last four weeks in the workplace. The general indicator was prepared by averaging the answers of the participants.

**Table 4:** Descriptive statistics and factor loadings in the confirmatory factor analysis of the Digital Transformation Stress Scale (DTSS) in Study 2 (N = 558).

	Item	M	SD	Factor loadings	SE	95% CI
DTSS	General indicator (average)	2.71	0.95	-	-	
Item 1	How often have you felt upset in connection with new ICT programs/systems?	2.86	0.93	.77*	.03	[.72, .83]
Item 2	How often have you felt irritated in connection with new ICT solutions which have affected your professional duties/tasks?	2.96	0.97	.77*	.03	[.72, .83]
Item 3	How often have you felt that you had no control over ICT changes connected with new procedures and your tasks?	2.83	0.96	.83*	.02	[.79, .88]
Item 4	How often have you felt annoyed because of new work tasks/rules in connection with system/program changes whose implementation you had no influence on?	2.86	0.91	.82*	.03	[.78, .87]
Item 5	How often have you felt that what was expected of you due to technological or IT changes was too much for you, to the point where you couldn’t cope with it?	2.37	0.96	.72*	.03	[.66, .77]
Item 6	How often have you felt that your competences and skills were insufficient to be proficient in new IT tools implemented at your workplace?	2.32	1.10	.65*	.03	[.59, .72]

*Note*: DTSS = Digital Transformation Stress Scale, SE = Standard Error, CI = Confidence Intervals

*p < .001

**The Perceived Stress Scale** (PSS-4) [41, 112] comprised of four items and was based on the Polish version of PSS [113, 114], modified to relate to general stress at work [114]. Participants were asked to describe their feelings and thoughts related to their professional work during the last month using a five-point scale where 1 = *Never* and 5 = *Almost always*, e.g., “How often have you felt that you were unable to control the important things in your life at work?”.

**Self-assessment ICT skills inventory**—To assess specific ICT skills in different areas, we developed the 7 items of ICT skills self-assessment inventory based on The Digital Competence Framework for Citizens [115]. Firstly, participants were asked to estimate their ICT skills in the workplace in general, (“Please evaluate your computer skills in the workplace”), by indicating the answers on a 5-point scale where 1 meant *Basic level*—*limited to elementary functionality* and 5 meant *Very advanced level*—*programming*, *graphic processing*, *computer operation of machines*. There was also a possibility to mark the answer “I’m not using a computer at work”. Afterwards, respondents were asked to determine their skills in the listed area and their activity on the Internet as well. It was performed in a matrix of statements applied to different ICT skills, from using keyboard shortcuts and internet transfer to working in different programs commonly used in the workplace. Example statements are: “I can prepare a presentation in a dedicated program, I can choose the layout, background, template, charts, tables.”, “I can pay by an online bank transfer”. The responses evaluated their skills on a 5-point scale, where 1 means *very low skill level* and 5 means *very high skill level*. The reliability of the Self-assessment ICT inventory was high (Cronbach’s alpha = .88).

**Digital transformation processes at the workplace**–a one-item question: “Are there any implementation projects (IT) currently being carried out in the organization where you work or study, which affect your work or your activities?”. Respondents have been asked to indicate an answer among “*Yes*, *there are”*, “*No*, *there are not”*, “*I do not know”* and “*Not applicable”*.

#### COVID– 19 impact assessment

To assess the impact of the COVID-19 pandemic on the participants’ professional life, we added a series of questions related to COVID-19, i.e., "Has the COVID-19 pandemic impacted your professional life?” Respondents selected answers between *Yes* and *No*. When they indicated *Yes*, this answer was followed by a few questions to specify this impact. Respondents could select multiple answers from the list like "I used to work more before COVID-19 pandemic”, “I started working remotely and it was something new for me", “I lost my job”, “I have gained a lot of new ICT skills (Information and Communication Technologies)”. There was also a possibility to enter their own statement describing the COVID-19 pandemic impact on the professional life of the participant.

#### Socio-demographic information

Participants were asked to indicate their age range, seniority range, gender, education level, occupation, and position in their current job.

### Results

#### Descriptive statistics

We again started our analysis from descriptive statistics, and we present means and standard deviations for all items and the general indicator of Digital Transformation Stress scale in Table 4. As in Study 1, the general level of digital transformation stress was moderate, with the value of mean close to the middle point of the scale, with variability close to one point of the scale.

#### Factorial structure and reliability

In this study, we again verified the unidimensional structure of DTSS using confirmatory factor analysis (CFA) in structural equation modelling approach in Mplus, version 8.2 [116]. Due to non-normality of the variables, we used Maximum Likelihood Robust (MLR) approach [117–119]. First, an exploratory analysis of the data is presented with descriptive statistics and correlations to evaluate the quality of the data. Then, a confirmatory factor analysis (CFA) was conducted. We specified one factor model loaded by all six items of the DTSS scale. We used modification indices to improve the preliminary model. The final model was evaluated using fit indices following Kline’s (57) recommendations, therefore we present Root Mean Square Error Approximation (RMSEA), Standardized Root Mean Square Residual (SRMR), the Comparative Fit Index (CFI) and the Tucker-Lewis Index (TLI) as well as the general fit based on χ^2^ test of model fit and its significance (*p*). We adopted widely recommended cut-off values indicative of an adequate model fit to the data, respectively: RMSEA and SRMR < .06 and < .08, CFI and TLI >.95 and >.90 [118].

The CFA analysis indicated that the preliminary model was not well fitted to the data χ^2^ (9) = 97.72, *p* < .001, CFI = .91, TLI = .85, RMSEA = .133, *p* = .001, 90% CI.[.110, .157], SRMR = .043. Following suggestions based on modification indices, we added covariance between two items with similar wordings–item 5 and item 6. This modified model was well fitted to the data χ^2^ (8) = 14.90, *p* = .061, CFI = .99, TLI = .99, RMSEA = .039, *p* = .678, 90% CI.[.001, .070], SRMR = .014. All factor loadings significantly loaded to one factor. Covariance between item 5 and item 6 equals .48. All factor loadings are presented in Table 4. Therefore, it can be concluded that the Confirmatory Factor Analysis confirmed a unidimensional structure of the DTSS scale.

#### Items difficulty and discrimination

Psychometric assessment of the items discrimination and difficulty was conducted using Item Factor Analysis in Item Response Model approach [120]. The current gold standard of estimation for IFA models is marginal maximum likelihood (MML) [121]. However, this estimation method in small samples does not provide information about global and absolute fit. Instead, we conducted assessment of model fit with weighted least squares estimator using mean and variance-corrected χ^2^ (WLMSV). Similarly to CFA, we used CFI, TLI, RMSEA, SRMR and χ^2^ test as measures of model fit with cut-off values described above to evaluate the goodness of the fit. Following the A two parameter model exhibited quite good fit measured by CFI = .963, TLI = .939 and SRMR = .036, but unacceptable fit by χ^2^ test of absolute fit χ^2^ (9) = 329.722, p < .001, and RMSEA = .253 [CI = .230, .276; p < .001]. A reduced model in which with one parameter fit significantly worse DIFFTEST(5) 24.211 p < .001. Thus, the original model was retained for further examination using MML estimation instead.

Model parameters obtained using MML and a logit link are shown in Table 5, which includes IFA item parameters (thresholds and loadings) as well as their Item Response Theory (IRT) analogous parameters of item discrimination and difficulty.

**Table 5:** Item loadings, thresholds, discrimination, and difficulty parameters for all items of the Digital Transformation Stress Scale (DTSS) in Study 2 (N = 558).

	**IRT Parameters**									
Item:	Discrimination (a)		Difficulty (b_1_)		Difficulty (b_2_)		Difficulty (b_3_)		Difficulty (b_4_)	
	Est.	SE	Est.	SE	Est.	SE	Est.	SE	Est.	SE
item 1	2.679	0.201	-1.052	0.083	0.089	0.062	1.381	0.091	2.595	0.205
item 2	2.640	0.193	-1.098	0.085	0.070	0.062	1.223	0.083	2.108	0.140
item 3	3.236	0.253	-1.114	0.080	-0.014	0.059	1.290	0.082	2.309	0.163
item 4	3.340	0.262	-1.008	0.077	0.148	0.058	1.231	0.079	2.138	0.141
item 5	2.681	0.209	-0.739	0.074	0.322	0.062	1.579	0.102	2.536	0.196
item 6	2.237	0.175	-0.826	0.082	0.375	0.067	1.649	0.114	2.837	0.242
	**IFA Parameters**									
Item:	Loading		Threshold (y > 1)		Threshold (y > 2)		Threshold (y > 3)		Threshold (y > 4)	
	Est.	SE	Est.	SE	Est.	SE	Est.	SE	Est.	SE
item 1	2.679	0.201	-2.820	0.229	0.239	0.166	3.701	0.257	6.952	0.519
item 2	2.640	0.193	-2.899	0.229	0.185	0.164	3.229	0.233	5.565	0.357
item 3	3.236	0.253	-3.604	0.296	-0.045	0.191	4.175	0.311	7.472	0.549
item 4	3.340	0.262	-3.366	0.290	0.494	0.197	4.112	0.311	7.141	0.511
item 5	2.681	0.209	-1.980	0.198	0.862	0.172	4.232	0.289	6.800	0.508
item 6	2.237	0.175	-1.847	0.175	0.838	0.152	3.688	0.243	6.347	0.489

It can be seen that all the items have similar discrimination parameters and it can be assumed that they similarly discriminate participants with different levels of latent trait. Also, threshold parameters for all items have quite similar values with a similar spread across values of latent variable.

**Fig 1:**
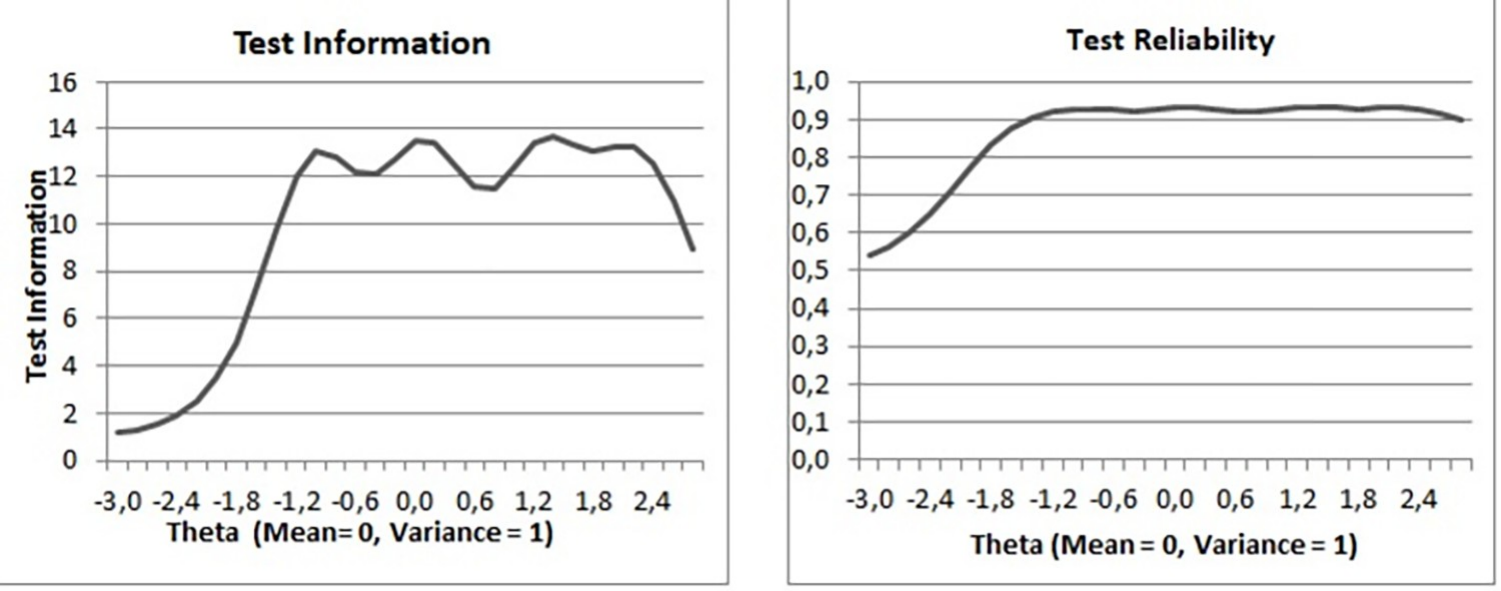
Test information function and test reliability of the Digital Transformation Stress Scale (DTSS) in Study 2 (N = 558).

Fig 1 (left panel) displays the test information function. As can be seen in that plot, the scale lacks measurement precision with theta falling below -2 but has high precision in moderate and high values of the latent variable. Overall, the scale yields precise measurement for individuals with moderate to high DTS levels and relatively imprecise measurement for individuals with low DTS levels. Additionally, in Fig 1 (right panel) we present test information for ease of interpretation converted into a traditional measure of reliability that ranges from 0 to 1. It shows that test reliability is higher than .80 for latent variable values ranging from -1.8Z, which is almost 2 standard deviations below the mean. It means that for almost 95% of population the measurement reliability is higher than .80 and for almost 91% is .90 or higher. Importantly, reliability is high for highest levels of digital transformation stress.

**Fig 2:**
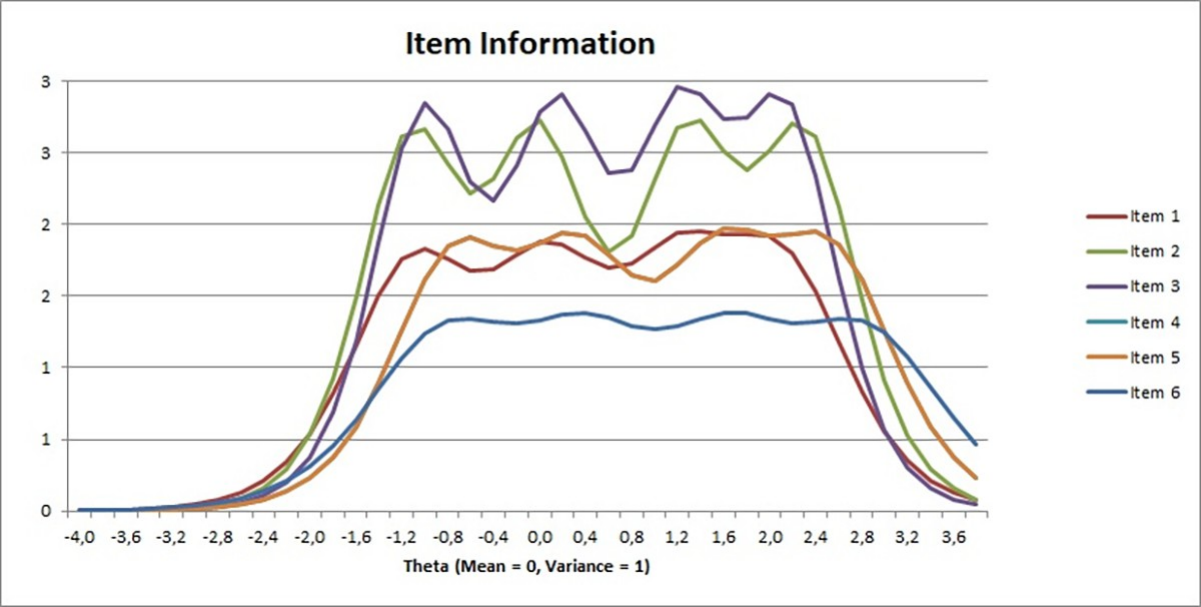
Items information functions of the Digital Transformation Stress Scale (DTSS) in Study 2 (N = 558).

We also present items information curves in Fig 2. As can be seen in Fig 2, one of the curves is a bit lower than others, namely the one for item 6. This indicates that overall degrees of measurement precision for this item are also relatively lower than for the rest of items. The highest plots are obtained for items 3 and 4, indicating higher measurement precision. Inspection of the item characteristics curves for all items (see S1 Fig) revealed that the pattern of curves is similar for all items, with a slightly higher curves for category 1 and category 5, and smaller for middle categories of responses. This may suggest that extreme categories of the response scale are more informative than the middle ones.

To test the reliability, we calculated Cronbach’s Alpha in IBM SPSS Statistics 27.0. The reliability was high (Cronbach’s Alpha α = .90) and it was similar to the results obtained in the first study.

#### Convergent validity

To present the convergent validity, we tested the association between general stress at work, measured with the Perceived Stress Scale (PSS) [41, 114] and stress of digital transformation, assessed with Digital Transformation Stress Scale. As predicted, we found a moderate and positive correlation between general stress in the workplace and DTSS with Pearson’s *r* = .45, *p* < .001. Employees with a higher level of digital transformation stress indicated a higher level of general stress at work. As predicted, indicators of general stress and digital transformation stress shared a rather moderate percentage of common variance (R^2^ = .20) and, therefore, they can be identified as separate constructs.

#### Divergent validity

*Ongoing IT implementation*. We expected that employees working in organizations with an ongoing digital transformation process should present higher digital transformation stress than those working in organizations with no digital transformation. Thus, we compared the level of digital transformation stress in these two groups using Student’s *t* test for independent samples. The results revealed that the level of DTS in employees working in organizations who implemented IT solutions was higher (*M* = 2.45, *SD* = 0.81) than for employees working in organizations without ongoing implementation (*M* = 2.29, *SD* = 0.79; *t*(426) = 2.14; *p* < .001, Cohen’s *d* = .21).

*COVID-19 impact*. The majority of participants (61.8%) stated that COVID-19 affected their professional lives, and we observed a significant difference in the level of DTS between the group of participants who did state that COVID-19 impacted their professional lives (*M* = 2.49, *SD* = 0.78) in comparison to participants who did not state that COVID-19 modified their working conditions (*M* = 2.24, *SD* = 0.79; *t*(556) = 3.72; *p* < .001, Cohen’s *d* = .32).

*ICT skills*. We verified if self-assessment of ICT skills is the factor of DTS. Thus, we examined the correlation between the DTSS and self-assessment of ICT skills inventory. For the entire sample (N = 558), we observed no correlation between two indicators: *r* = -.04, *p* = .31. Additionally, we calculated the correlation between ICT skills and digital transformation stress only for those participants who declared that there is an ongoing IT solutions implementation in their organization (see Table 6). There was significant negative correlation between self-assessment skills and digital transformation stress in the group of participants who reported that there was ongoing implementation IT process in their organisation. However, this correlation was very weak. Such a correlation was not present in the group of employees who declared that there is no such implementation in their organisation.

**Table 6:** Correlations among general stress, digital transformation stress and ICT skills in groups of employees working in organizations with and without ongoing digital solution implementation in Study 2 (N = 558).

Group		Pearson’s *r* coefficients (N = 558)	1.	2.
Ongoing implementation = *Yes*	1.	Digital Transformation Stress (DTSS)	-	
	2.	General Stress (PSS-4)	.43**	-
	3.	Self-assessment ICT skills	-.16*	-.17**
Ongoing implementation = *No*	1.	Digital Transformation Stress (DTSS)	-	
	2.	General Stress (PSS-4)	.48**	-
	3.	Self-assessment ICT skills	.03	-.15*

Note

** *p* < 0.01

* *p* < 0.05. ICT = information and communication technologies

### Discussions and conclusions

The first goal of Study 2 was to examine whether we could confirm the factorial structure of DTSS found in Study 1 in a new sample of professionally active participants. The second goal of the study was to evaluate the difficulty of the items and their discrimination, and the third goal was to evaluate the convergent and divergent validity of DTSS. Firstly, the results of Confirmatory Factor Analysis confirmed the unidimensional structure of Digital Transformation Stress Scale, observed in Study 1. Secondly, the level of difficulty and discrimination evaluated in IRT approach was similar for all items and the whole scale had a good reliability for moderate and high level of measure latent variable. Thirdly, confirming our predictions, the correlations coefficients indicated that the level of DTS was positively but rather moderately related with general, perceived stress in the workplace. As predicted, the correlation between the DTS and self-assessment ICT skills was also weak, confirming that this type of stress, in contrast to technostress, does not stem from lack of IT skills.

We also tested the role of COVID-19 impact, as perceived by the employees. Most of the participants stated that COVID-19 impacted their professional lives and as predicted, we observed significant differences in digital transformation stress level between two groups of participants: those who stated that COVID-19 impacted their professional life had an elevated level of DTS in comparison to those who felt unimpacted by COVID-19. The same pattern of results was observed in the presence of the factor of ongoing ICT solutions implementation processes. Employees working in organisations with ongoing implementations had a higher level of digital transformation stress in comparison to those employees who worked in the organisation without such implementations. Taken together, these results can be interpreted as prior evidence that acceleration of DT can be the source of DTS.

## General discussion

The main aim of our research was to psychometrically evaluate the Digital Transformation Stress Scale. Based on a comprehensive literature review, we defined DTS as employees’ stress related directly to the DT process itself, as employees’ response to the process and the mode of DT project management. Following this definition, we constructed a six-item Digital Transformation Stress Scale to evaluate the level of digital transformation stress in the workplace. In line with theoretical assumptions, we identified digital transformation stress as one of the sources of general stress at work and we assumed that these two constructs are related, though not the same. Therefore, we predicted that a significant, but rather moderate, correlation on these two constructs of our data would be found. We also distinguished digital transformation stress from technostress, by pointing out the limited role of perceived ICT skills in elevating digital transformation stress. We assumed that the level of ICT skills in employees should not be strongly related to digital transformation stress. We also predicted that digital transformation stress should be higher in employees working in organizations that had been implementing digital solutions. As the COVID-19 pandemic situation strongly impacted the acceleration of DT [15, 24, 32, 122], we also anticipated that those workers who believed to be strongly impacted by the pandemic would present a higher level of digital transformation stress.

The results of our studies generally presented Digital Transformation Stress Scales as a valid tool to evaluate the level of digital transformation stress. The results of our exploratory and confirmatory factor analyses as well as IRT approach confirmed the one-dimensional structure of DTSS and its good reliability in both studies. In line with our predictions, we observed no correlation between DTSS and self-assessed ICT skills in quite a numerous and diverse sample in Study 2, even when we limited participants to those whose organizations were undergoing a digital transformation process.

Although there was a significant difference in DTSS levels between the two groups, it was relatively small. This pattern of results can stem from several processes. Firstly, it is the way of introducing ICT solutions that may have significant impact on the level of perceived stress [93, 88, 122], stress resulted from digital transformation. However, in our study participants were asked only about the presence of the digital implementation and did not evaluate the quality of their own project and digital transformation management.

Secondly, according to the CoR and the JD-R theories, stress is the psychological response that arises when job demands (availability, workload and lack of control) and resources (organisational, technical support and social support) are imbalanced. Although the DTS scale includes items related to the assessment of personal resources, such as the sense of control or competence, they are of an emotional nature. Therefore, we see the need to compare the measurements of the perceived stress of digital transformation with a scale examining attitudes to digital transformation, consisting of cognitive, emotional, and behavioural dimensions. Similarly, this transactional nature of stress was unfortunately not captured precisely by the measure of ICT implementation used in our study, as we did not evaluate both job demands and resources in ongoing digital transformation process. In a further study, we would like to address this limitation by exploring the interaction of ICT demands (e.g. ICT hassles, ICT availability, ICT workload and ICT lack of control [14, 15]) with employees’ resources (e.g. like self-efficacy [45, 123] or ICT support [16].

Finally, we believe that the COVID-19 pandemic, being the global factor, had forced rapid digital transformation and changes in almost all organisations. Although switching to the remote work arrangement and technical adaptations processes as well as adaptation of the IT infrastructure could not be perceived as the ongoing implementation project per se, these processes could impact employees in the same way as IT solution projects and digital changes. Consequently, all the above may increase the digital transformation stress level. Because of that, observed differences between workers in organisations with ongoing implementations and those without implementations did not differ strongly in terms of DTS. All of these processes may explain why differences in DTS between these two groups of employees were not more noticeable.

Summarizing, our studies showed that DTSS may serve as a reliable instrument in measuring employees’ perceived DT stress in response to the specific process of DT in organizations. Being a short scale, it may also be useful as an indicator of DTS change after introducing psychological interventions aimed to reduce this type of stress.

### Limitations and future research directions

The present study has several limitations which need to be discussed. Firstly, respondents, especially in the first study, were invited mainly via social media, particularly through LinkedIn, and this may seriously limit the generalizability of our findings. The online way of conducting the study might have reduced the number of participants who were very strongly affected by DTS. Therefore, further research on the role of different modes of survey administration are necessary to assess the influence of this factor on the level of DT stress observed at the workplace

Secondly, as stated above, some limitation of our research includes the measure used to identify organizations with ongoing digital transformation process. As it was based only on self-reports provided by employees, it might not precisely capture the higher level of DTS demands related to implementations. It would be very interesting to select organizations just as they start the process of digital implementation and evaluate changes in the level of digital transformation stress of their employees longitudinally. Such research design would enable us to provide more reliable information about its causal relationship between employees’ demands, resources and stress.

Thirdly, as the preliminary set of items was rather short, quantitative research on DTS is required to identify such symptoms of DTS that may differentiate it from job stress and technostress. The following construction process may also include additional dimensions, related to secondary appraisals of stress evoked by change, identified in applied research on organisational change. As Olafsen et al. [124] proposed, an important factor linked to individuals’ reaction to change is change commitment, which can be described using three components: affective, normative, and calculative [125, 126]. These dimensions allowed to predict individual readiness to change, namely change self-efficacy, and are perceived as significant predictors of commitment to organisational change. These personal characteristics of the employees, perceived as a potential resource, may also be related to digital transformation stress.

Finally, the phenomenon of DTS is conceptually larger than only emotional dimension therefore further research on digital transformation stress should be expected. We assess that deeper analysis on digital transformation stress and digital transformation attitudes should be conducted, taking into account the multidimensional construct of stress i.e., cognitive, behavioural and emotional.

Further work should also examine how strongly DT stress impacts different work outcomes such as work commitment, job satisfaction, and burnout of employees. Additionally, more research is needed to examine the variables that allow to predict the level of DTS e.g., attitudes toward digital implementation, perceived work demands. This research should also be conducted after the COVID-19 pandemic, when DT processes will be simpler to plan and properly implemented in organizations.

### Theoretical and practical implications

The general aim of our studies is to propose a self-descriptive and easy-to-administer measurement tool aimed to identify highly stressed employees who would benefit from specific psychological interventions reducing DT stress. The DTSS possesses satisfactory psychometric properties but is also attractive for reasons of cost and time effectiveness, especially in automated screening systems that might be constructed using this scale [55, 89]. Being a rather brief scale, DTSS is more user-friendly and may have a higher response rate in comparison to more elaborated measures.

In the future, DTS scale may become a substantial part of an automated system consisting of three hierarchical elements: a) preliminary screening, based on a qualitative analysis of help-desk tickets sent by employees [89], b) evaluation of the level of DT stress based on the DTSS [55] to identify highly stressed employees, c) invitation to take part in a psychological intervention, the effectiveness of which we presented in our previous study [55]. Designing and testing such systems may play a crucial role for preserving psychological well-being of the employees, especially when situation demands a rapid DT implementation at the workplace.

Moreover, in project management area [127, 128], the Digital Transformation Stress Scale may be used to compare different project management methodologies, typically used in implementing digital transformations, e.g., the waterfall technique with more modern agile techniques [128–131]. The waterfall, being more traditional, includes a set of techniques used for planning, estimating, and controlling activities [90, 96]. Using a simplification, it strictly separates implementation phases: the analysis phase, the implementation, and the testing phase. Often, final users–namely employees, are not involved in the initial stages but instead they start working with new solutions in the testing phase, very often under time pressure [131]. Agile technique is a more flexible [127, 129, 131, 132], iterative approach in which end-users are more actively taking part in the whole process and can gradually learn about and test new solutions [10, 127, 129]. Measurement of the perceived digital stress in key in these two project management methodologies. It allows to compare the level of digital transformation stress in different project phases and identify the phases in which the employees should be actively assisted. Therefore, using DTSS could be an efficient way to manage and improve the digital transformation process in organizations.

From the theoretical standpoint, DTSS can be used to further elaborate psychological antecedents (e.g., DT demands), and new sources of stress in various contexts e.g., at the workplace, in education, in the health-care systems—wherever the digital implementations are introduced in the hope of improving the effectiveness of the system. Research testing the associations between DTS and psychological well-being, burnout, disengagement is of great importance in understanding and evaluating the value of these costs.

### Conclusions

The COVID-19 pandemic has highlighted the importance of digitalization in many areas of business, accelerating the digital transformation process. Although the digital maturity of organizations and employees is increasing overall, there are considerable consequences of DT. The negative effects of DT on employees appear due to the process of introducing changes under time pressure, without proper planning, and gradual implementation [33, 133]. In consequence, we observe a strong impact on employees’ comfort of work and their stress related to the acceleration of digital changes in organizations [38, 26, 27]. In our studies we present a psychometric evaluation of Digital Transformation Stress Scale designed to assess the level of digital transformation stress at the workplace. Our results confirmed good psychometric properties of the DTSS, and this may enable researchers to address the root causes of DTS with proper guidelines and interventions. Evaluation of the DTS level may also help alleviate this type of stress by helping employees deal with perceived digital transformation stress. As we presented in our previous studies, DTSS can be successfully applied as s screening tool to identify employees who suffer from this type of stress, and also to assess the effectiveness of the psychological intervention offered to reduce DTS [134]. This may also be beneficial at the organizational level by supporting businesses which would benefit from improved efficiency, satisfaction, and well-being of their employees.

## Supporting information

S1 Fig Item characteristics curves for all items of Digital Transformation Stress Scale. (DOCX)

S1 Data (SAV)

S2 Data (SAV)
