# Supplementary figures and images for: Measuring digital transformation stress at the workplace–Development and validation of the digital transformation stress scale
Source: PLoS One. 2023 Oct 18;18(10):e0287223. doi: 10.1371/journal.pone.0287223 (PMC10584111; doi:10.1371/journal.pone.0287223)

S1 Fig.. Item characteristics curves for all items of Digital Transformation Stress Scale


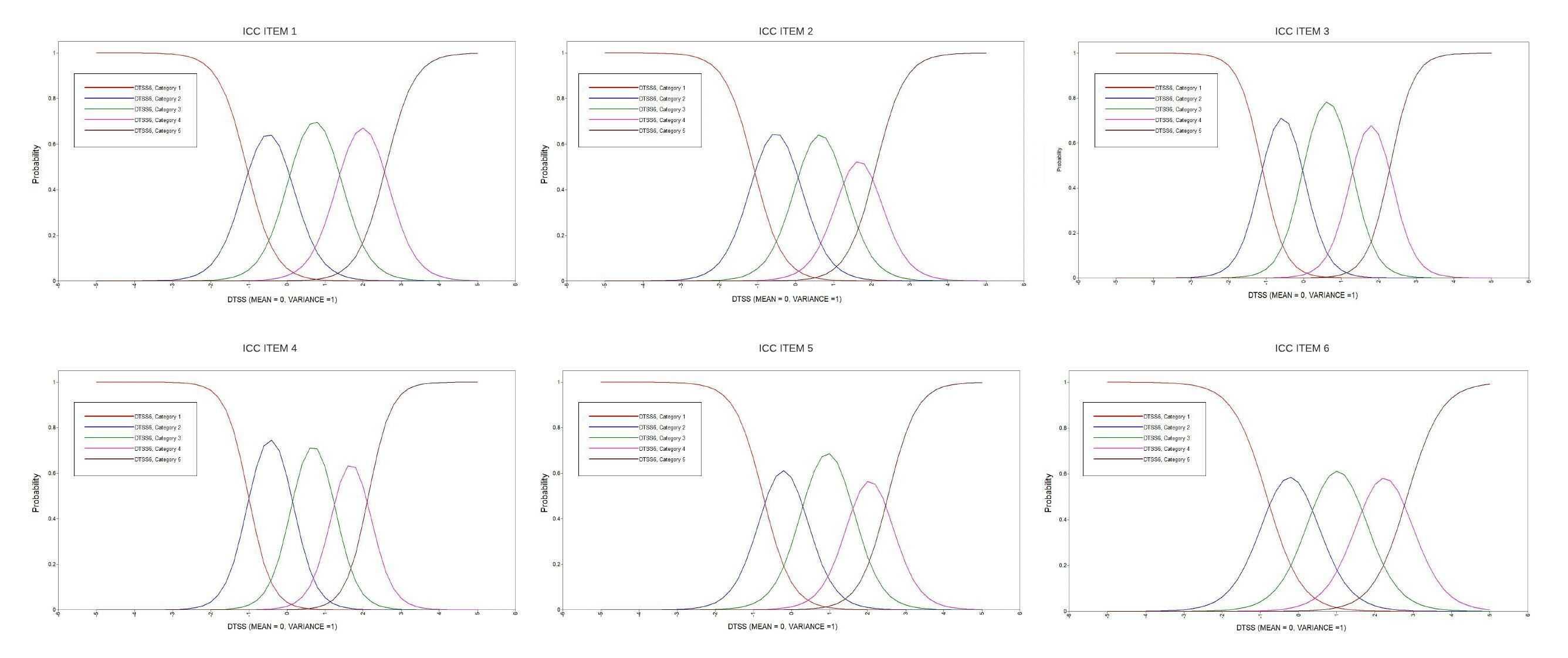

Supplement: S1 Fig — (DOCX) [file pone.0287223.s001.docx]
